# Peritonsillar abscess as a clinical sign of oropharyngeal cancer – a Swedish national cohort study

**DOI:** 10.1007/s00405-026-10235-7

**Published:** 2026-06-01

**Authors:** Eivind Gottlieb-Vedi, Giola Santoni, Sanna Toppila-Salmi, Antti Mäkitie, Shao-Hua Xie, Jesper Lagergren

**Affiliations:** 1 Upper Gastrointestinal Surgery, Department of Molecular Medicine and Surgery, Karolinska Institutet, and Karolinska University Hospital, Blombäcks väg 23, plan 4, Stockholm, 17177 Sweden; 2 Department of Otorhinolaryngology, University of Eastern Finland, Joensuu and Kuopio, Finland and Wellbeing services county of Pohjois-Savo, Kuopio, Finland; 3 Department of Allergology, Inflammation Center, Helsinki University Hospital, University of Helsinki, Helsinki, Finland; 4 Division of ENT diseases, Department of Clinical Science Intervention and Technology, Karolinska Institutet, Stockholm, Sweden; 5 Department of Otorhinolaryngology, Head and Neck Surgery, University of Helsinki and Helsinki University Hospital, Helsinki, Finland; 6 School of Cancer and Pharmaceutical Sciences, King’s College London, and Guy’s and St Thomas’ NHS Foundation Trust, London, UK

**Keywords:** Tonsil abscess, Tonsil cancer, Oropharyngeal neoplasm

## Abstract

**Purpose:**

Patients older than 40 years with peritonsillar abscess may routinely undergo follow-up to detect oropharyngeal cancer, but this approach lacks scientific evidence. This study aimed to assess the hypothesis that oropharyngeal cancer is not overrepresented in patients with peritonsillar abscess.

**Methods:**

This population-based, nationwide Swedish cohort study compared all patients diagnosed with peritonsillar abscess during 2005–2023 with an age-, date-, and sex-matched non-exposed group from the same cohort. Conditional logistic regression provided odds ratios (OR) with 95% confidence intervals (CI) for oropharyngeal cancer within 1 year after peritonsillar abscess diagnosis, or matching date. Except for the three matching variables, the OR were adjusted for tobacco smoking, alcohol overconsumption, and comorbidity in a multivariable model.

**Results:**

Oropharyngeal cancer was diagnosed in 30 patients out of 64,244 occurrences of peritonsillar abscess (0.05%), compared to 9 out of 642,440 individuals in the comparison group (0.0014%). All 39 oropharyngeal cancers occurred in individuals aged 40 or older. Peritonsillar abscess was associated with an increased 1-year risk of oropharyngeal cancer (adjusted OR 3.51, 95% CI 2.75–4.26). In substrata of age, the strongest association was observed for patients aged 60 years and above (adjusted OR 4.54, 95% CI 3.07–6.02), corresponding to 2.8 cases per 1000 patients, while the age group 40–59 years had an OR of 2.76 (95% CI 1.80–3.72).

**Conclusion:**

Peritonsillar abscess may indicate an underlying oropharyngeal cancer only in rare cases. Given the low incidence of oropharyngeal cancer after peritonsillar abscess, routine follow-up is questionable or may be limited to older patients.

## Introduction

Oropharyngeal cancer refers to a group of cancers of the tonsils, tongue base, soft palate, and posterior and lateral pharyngeal walls, affecting nearly 100,000 individuals globally each year [1, 2]. In Sweden, oropharyngeal cancer is the second most common head and neck cancer and has the fastest increasing incidence, currently accounting for approximately 450 incident cases annually [3]. The relative 5-year survival is around 70% after diagnosis [3]. The dominating histological subtype is squamous cell carcinoma, for which tobacco smoking, alcohol overconsumption, and infection with human papilloma virus are risk factors [4]. Main treatment options include surgery, with or without neck dissection, radiotherapy and chemoradiotherapy [5].

A peritonsillar abscess is located between the capsule of a palatine tonsil and the pharyngeal muscles, believed to be caused by bacterial infection of the tonsils or peritonsillar accessory salivary glands (Weber’s glands) [6, 7]. Whereas the mean age at diagnosis of oropharyngeal cancer is around 60 years, the incidence of peritonsillar abscess is highest in adolescents and young adults, followed by a decreasing incidence with older age [8, 9]. Patients aged ≥40 years are often followed up after the abscess has resolved in order to screen for oropharyngeal cancer by visual inspection and manual palpation. This practice is based on clinical experience that oropharyngeal cancers may present as peritonsillar abscess, as described in case reports and case series [10–13]. Higher level evidence on the topic is, however, lacking. This suggests an unmet need to investigate the association between peritonsillar abscess and oropharyngeal cancer in a well-designed study to address whether all or certain subgroups of patients benefit from routine clinical follow-up to exclude oropharyngeal cancer after treatment for peritonsillar abscess.

This nationwide Swedish study aimed to examine the hypothesis that oropharyngeal cancer is not overrepresented in patients with peritonsillar abscess.

## Methods

### Design

This was a population-based cohort study that encompassed all Swedish residents using certain drugs for indications within the alimentary tract and metabolism, platelet aggregation inhibitors, cardiovascular system, genito urinary system and sex hormones, endocrine therapy, antibacterials for systemic use, anti-inflammatory and antirheumatic products, analgesics and nitroimidazole derivatives between 1st July 2005 and 31st December 2023. The incidence of oropharyngeal cancer within one year following a peritonsillar abscess was compared with the corresponding incidence in a matched comparison group.

### Cohort

The study was based on an updated version of a nationwide cohort (The Swedish Prescribed Drugs and Health Cohort [SPREDH]), which has been described in detail elsewhere [14]. In brief, the updated cohort includes 9,454,340 individuals who had ever dispensed medications listed in the section above during the study period, according to the Swedish Prescribed Drug. This number corresponds to the vast majority of the entire Swedish population (10.5 million people, including children). The Drug Registry contains data on all prescribed and dispensed medications from all pharmacies in Sweden, including Anatomical Therapeutic Chemical (ATC) codes and dates [15]. Using the personal identity numbers assigned to each Swedish resident at birth or upon immigration, each individual’s data were linked to the Swedish Patient Registry, the Swedish Cancer Registry, and the Swedish Cause of Death Registry. The Swedish Patient Registry contains information on all inpatient care and specialized outpatient care in Sweden since 1987 and 2001, respectively, with nearly 100% completeness, and it provided information on diagnosis and date of peritonsillar abscess, diagnoses associated with tobacco smoking and alcohol overconsumption, other comorbidities, as well as age and sex of each participant [16]. The Swedish Cancer Registry, which contains data on all malignant tumors diagnosed in Sweden and has more than 96% overall completeness, provided information on date and diagnosis of oropharyngeal cancer [17]. The Swedish Cause of Death Registry is 100% complete for date of death and was used to censor participants who died before the end of follow-up.

### Exposure

Individuals with a diagnosis of peritonsillar abscess (10th version of the International Classification of Diseases [ICD-10] code J36) were included if the diagnosis was reported in combination with a prescription of an antibiotic drug (ATC-code J01 or P01AB01) within 30 days of hospital discharge or out-patient visit. The prescription of antibiotics was included close to the time of peritonsillar abscess diagnosis to ensure higher diagnostic accuracy. Antibiotics can only be dispensed through a prescription in Sweden [18]. Additional peritonsillar abscesses were included in the same individual if: (1) no peritonsillar abscess was defined within 30 days before, and (2) it was the last such diagnosis occurring within the follow-up period of 1 year, or (3) the follow-up period of one year had passed. Index date for exposed people was the time of antibiotic prescription relative to the included peritonsillar abscess diagnosis. For each peritonsillar abscess event, 10 people were randomly selected from the SPREDH cohort matched by age (same year) at index date, index date, and sex. Matching was conducted to make data handling more manageable whilst ensuring maximum power. The unexposed matched individuals had no history of peritonsillar abscess before the matching date. In both the exposed and non-exposed groups, individuals with a diagnosis of oropharyngeal cancer at or before the index date were excluded.

### Outcome

The outcome was oropharyngeal cancer (7th version of ICD [ICD-7] code 145) occurring within one year from index date. Follow-up time was limited to one year in order to increase the likelihood of oropharyngeal cancers being prevalent as underlying etiology (but undiagnosed) of the peritonsillar abscesses.

### Statistical analysis

The statistical analyses followed a detailed, priori-defined study protocol. Baseline variables were presented descriptively as median values with interquartile ranges (IQR) for continuous variables and as absolute numbers with frequencies for categorical variables. Odds ratios (OR) with 95% confidence intervals (CI) were calculated using conditional logistic regression comparing the 1-year incidence of oropharyngeal cancer in the peritonsillar abscess group with the matched non-exposed group. Logistic regression was selected as time-to-event within the follow-up period was not deemed relevant, and to decrease possible bias due to earlier detection of oropharyngeal cancer in the peritonsillar abscess group. The results from a crude model were not adjusted for any variables, but were matched for age, index date, and sex. The results from a multivariable model were additionally adjusted for comorbidity (Charlson comorbidity index score, 0, 1, or ≥ 2) [19], tobacco smoking (smoking-related diseases, yes or no), and alcohol overconsumption (alcohol-related diseases, yes or no). All confounders were assessed at index date. Alcohol-related diseases included diagnosis codes suggested in a previous publication [20]. Smoking-related diagnoses included the following diagnosis codes: 490–492, 494, 496, and 305B (ICD-9), and J40-J44, J47, Z72.0, F17, Z71.6, and T65.2 (ICD-10).

Stratified analyses were conducted by including an interaction between the exposure and each variable, extracting OR with the corresponding 95% CI from each substratum for the following four variables: age (40–59 and ≥60 years), sex (male and female), calendar year (≤ median and > median), and comorbidity (Charlson comorbidity index score 0 and ≥ 1). The statistical power was not sufficient to perform stratified analyses for the variables smoking- or alcohol-related diagnoses. The proportion of oropharyngeal cancer incidence in each group and within subgroups was presented as the number of cancers per 1000 participants, with 95% CI calculated by the Clopper-Pearson Exact method.

## Results

### Patients

Among 9,454,340 unique individuals in the cohort, 64,244 peritonsillar abscesses were identified in 56,668 unique patients, of whom 49,994 (88.2%) had one abscess, 5,911 (10.4%) had two abscesses, and 763 (1.3%) had ≥ 3 peritonsillar abscesses. Selected overall characteristics of the exposed individuals are presented in Table 1. Among the patients with abscesses, the median age was 30 years, and 69.2% were younger than 40 years. The sex distribution was equal, and most patients (85.1%) had no comorbidity. Smoking- and alcohol-related diseases were found in 1.3% and 3.3%, respectively. As presented in Table 2, which describes the characteristics of individuals included in the logistic regression, patients with peritonsillar abscess had more comorbidities than those in the non-exposed group (Charlson comorbidity index score ≥1 in 35.9% versus 26.7%) and a higher prevalence of alcohol-related diseases (5.1% versus 2.8%). Death before end of follow-up occurred in 7.7% in the abscess group and in 0.8% in the non-exposed group.

**Table 1:** Characteristics of patients with peritonsillar abscess in Sweden during 2005–2023

Characteristics	Peritonsillar abscess Number (%)
Total	64,244 (9.1)
Age in years	
< 40	44,447 (69.2)
40–59	13,063 (20.3)
≥60	6,734 (10.5)
Sex	
Male	33,430 (52.0)
Female	30,814 (48.0)
Calendar year	
2005–2014	32,603 (50.8)
2015–2023	31,641 (49.3)
Charlson Comorbidity Index score	
0	54,656 (85.1)
≥1	9,588 (14.9)
Smoking-related disease	
No	63,492 (98.8)
Yes	841 (1.3)
Alcohol-related disease	
No	62,004 (96.5)
Yes	2,136 (3.3)
Oropharyngeal cancer	30 (0.05)

**Table 2:** Characteristics of patients with peritonsillar abscess and age-, date-, and sex-matched individuals without peritonsillar abscess in Sweden during 2005–2023

Characteristics	No peritonsillar abscess	Peritonsillar abscess
	Number (%)	
Total	390 (90.9)	39 (9.1)
Age in years		
40–59	180 (46.2)	18 (46.15)
≥60	210 (53.9)	21 (53.9)
Sex		
Male	260 (66.7)	26 (66.7)
Female	130 (33.3)	13 (33.3)
Calendar year		
2005	0	0
2006–2014	200 (51.3)	20 (51.3)
2015–2023	190 (48.7)	19 (48.7)
Charlson Comorbidity Index score		
0	286 (73.3)	25 (64.1)
≥1	104 (26.7)	14 (35.9)
Smoking-related disease		
No	386 (99.0)	38 (97.4)
Yes	4 (1.0)	1 (2.6)
Alcohol-related disease		
No	379 (97.2)	37 (94.9)
Yes	11 (2.8)	2 (5.1)
Death before end of follow-up	3 (0.8)	3 (7.7)

### Frequencies of oropharyngeal cancer

Among the patients with peritonsillar abscess, 30 (0.05%) developed oropharyngeal cancer during the 1-year follow-up. Twenty-nine (97%) of them were squamous cell carcinomas, and 26 (87%) occurred in the tonsil. In the non-exposed group, nine (0.0014%) patients developed oropharyngeal cancer during the 1-year follow-up, and all were tonsillar squamous cell cancer. All cancers occurred in participants aged > 40 years.

### Absolute risk of oropharyngeal cancer after peritonsillar abscess

The overall incidence of oropharyngeal cancer among the patients with peritonsillar abscess was 0.5/1000 patients (95% CI 0.3–0.7), and it was higher in patients aged ≥60 (2.8/1000 patients [95% CI 1.8–4.4]) than in those aged 40–59 years (0.8/1000 [95% CI 0.5–1.5]) (Table 3). Higher incidence rates were observed in participants with comorbidities, smoking-related diagnoses, and alcohol-related diagnoses, compared with those without (Table 3).

**Table 3:** Number of diagnosed oropharyngeal cancers per 1000 individuals, with 95% confidence intervals, in patients with peritonsillar abscess and in age-, date-, and sex-matched individuals without peritonsillar abscess in Sweden during 2005–2023

	No peritonsillar abscess	Peritonsillar abscess
	Oropharyngeal cancer /1000 individuals (95% confidence interval)	
Total	0.01 (0.01–0.03)	0.5 (0.3–0.7)
Age in years		
40–59	0.05 (0.03–0.11)	0.8 (0.5–1.5)
≥60	0.03 (0.01–0.12)	2.8 (1.8–4.4)
Sex		
Male	0.02 (0.01–0.04)	0.6 (0.4–0.9)
Female	0.01 (0.002–0.03)	0.4 (0.2–0.6)
Calendar year		
2005–2014	0.01 (0.01–0.03)	0.5 (0.3–0.8)
2015–2023	0.02 (0.01–0.04)	0.4 (0.3–0.7)
Charlson Comorbidity Index score		
0	0.02 (0.01–0.03)	0.3 (0.2–0.5)
≥1	0.01 (0.001–0.07)	1.3 (0.7–2.2)
Smoking-related disease		
No	0.01 (0.01–0.03)	0.5 (0.3–0.7)
Yes	0	1.3 (0.2–9.4)
Alcohol-related disease		
No	0.01 (0.01–0.03)	0.4 (0.3–0.6)
Yes	0	1.3 (0.4–4.1)

### Relative risk of oropharyngeal cancer

Peritonsillar abscess was associated with an increased risk of oropharyngeal cancer (adjusted OR 3.51, 95% CI 2.75–4.26) (Table 4). The OR was 2.76 (95%, CI 1.80–3.72) in patients aged 40–59 years and 4.54 (95% CI 3.07–6.02) in patients aged ≥ 60 years. The stratified analyses showed slightly higher point estimates in women than in men and in those with comorbidity than in those without, while the risk estimates were similar across calendar-year strata (Table 4).

**Table 4:** Risk of oropharyngeal cancer in patients with peritonsillar abscess compared with age-, date-, and sex-matched individuals without peritonsillar abscess in Sweden during 2005–2023

Model	No peritonsillar abscess	Peritonsillar abscess
	Odds ratio (95% confidence interval)	
Crude	1.00 (reference)	3.51 (2.76–4.25)
Adjusted^1^	1.00 (reference)	3.51 (2.75–4.26)
Subgroups^2^		
Age in years		
40–59	1.00 (reference)	2.76 (1.80–3.72)
≥ 60	1.00 (reference)	4.54 (3.07–6.02)
Sex		
Male	1.00 (reference)	3.30 (2.42–4.17)
Female	1.00 (reference)	4.01 (2.48–5.53)
Calendar year		
2006–2014	1.00 (reference)	3.71 (2.60–4.82)
2015–2023	1.00 (reference)	3.30 (2.26–4.33)
Charlson Comorbidity Index score		
0	1.00 (reference)	3.11 (2.21-4.00)
≥ 1	1.00 (reference)	4.77 (2.48–7.05)

^1^Adjusted for Charlson comorbidity index score, smoking-related diseases, and alcohol-related diseases

^2^Adjusted for the above, with the addition of an interaction term between the exposure and variable of interest

## Discussion

The results of this first analytical study on the potential malignant etiology of peritonsillar abscesses showed an increased relative risk of oropharyngeal cancer among these patients, especially in older individuals, but the absolute risk was still low.

Key strengths of the study include the population-based design and the high quality and completeness of the prospectively collected data on the exposure, confounders, and outcome. A potential weakness is that peritonsillar abscesses treated solely in primary healthcare were not included, as the Patient Registry captures only specialised healthcare (in- and out-patient). However, in Sweden these patients are managed in specialised healthcare due to the need for drainage [21]. As such, most cases are likely included as supported by the study’s observed annual incidence of peritonsillar abscesses, which is in line with previously reported data [22, 23]. It cannot be ruled out that the observed difference in incidence between the groups, at least to some extent, is due to detection bias, i.e. that current clinical practice with follow-up after peritonsillar abscess makes this group more likely to be diagnosed with oropharyngeal cancer. This might explain why oropharyngeal cancer was more common in patients with peritonsillar abscess, overestimating the strength of the observed association. However, the use of logistic regression, rather than time-to-event analysis, was chosen to decrease this risk. The study covers the COVID-19 pandemic years, during which previous research suggests a decrease in diagnosed patients and delayed diagnosis [24]. The definition of peritonsillar abscess, which requires both diagnosis and antibiotic prescription, counteracted misclassification. Confounding cannot be completely excluded, despite the fact that the results were controlled for several variables by matching or adjustment. Moreover, tobacco smoking and alcohol overconsumption were assessed by diagnoses closely associated with these exposures rather than directly from the study participants. This could lead to residual confounding of these variables. However, these exposures are often under-reported, and the assessment of associated diseases may be more objective. Despite the large size of the cohort, the number of patients with oropharyngeal cancer was low, limiting the statistical power, especially in subgroup analyses. Information regarding infection with human papilloma virus (HPV) was not available, and it cannot be determined whether peritonsillar abscess associated cancers differ from the general oropharyngeal cancer population in HPV status. However, because HPV is not a known risk factor for peritonsillar abscess it does not represent a confounding variable for the current research topic and the lack of information for HPV status should thus not introduce any bias.

Previous evidence on the subject is scarce. Authors of a case series from England reviewed medical records of patients diagnosed with peritonsillar abscess or cellulitis and found that 0.15% had been diagnosed with oropharyngeal cancer within two years of follow-up, all of which were tonsil cancer [25]. No comparison group was used in the study, but the age-standardised incidence rate of oropharyngeal cancer in the United Kingdom is 0.91 per 100,000 person-years [26]. The observed incidence of oropharyngeal cancer during the 1-year follow-up in the current study was approximately 0.05%. The lower incidence may be due to the shorter follow-up time in the current study, while the larger sample size contributed to greater precision in the estimates.

Despite the strongly increased relative risk of peritonsillar abscess and oropharyngeal cancer observed in the current study, the absolute risk of oropharyngeal cancer remained low. In the age group 40–59 years, one in 1,250 patients with peritonsillar abscess was diagnosed with oropharyngeal cancer. The corresponding number was 1 in 357 in patients aged 60 years and above with peritonsillar abscess. These numbers assume 100% sensitivity in identifying suspected cancerous tumours requiring further investigation, which is unlikely. Moreover, according to current Swedish clinical guidelines, an examination is recommended after the infection has resolved, but this might delay cancer diagnosis and attenuate potential gains from routine follow-up.

The results may serve to guide clinical decision-making in the soundness of routine follow-up of patients with peritonsillar abscess, with the aim of facilitating earlier detection of oropharyngeal cancer. The low incidence of oropharyngeal cancer in the present study raises the question whether this approach is meaningful, and whether follow-up should be limited to older patients than what is practice today.

In conclusion, peritonsillar abscess seems to be associated with an increased relative risk of oropharyngeal cancer, but the absolute risk is low. These results question the need for routine follow-up for cancer detection purposes in patients with peritonsillar abscess. A more tailored approach for certain high-risk patients may offer greater clinical value. However, before a recommendation can be given, the results need to be verified in other studies.

## References

[1] Hay A, Nixon IJ (2018) Recent advances in the understanding and management of oropharyngeal cancer. F1000Res 7. 10.12688/f1000research.14416.1 10.12688/f1000research.14416.1 PMC6117847 30228861

[2] Bray F, Laversanne M, Sung H et al (2024) Global cancer statistics 2022: GLOBOCAN estimates of incidence and mortality worldwide for 36 cancers in 185 countries. CA Cancer J Clin 74(3):229–263. 10.3322/caac.21834 38572751 10.3322/caac.21834

[3] Centres CoRC (2020) The Swedish head and neck cancer register, SweHNCR, Presentation in English [Internet]. Göteborg: Regionalt cancercentrum väst, Västra sjukvårdsregionen [cited 11 Feb 2024]. Available from: https://cancercentrum.se/download/18.3de350c219545ea25bd220e1/1741017690273/presentation-in-english-swehncr.pdf

[4] Lechner M, Liu J, Masterson L, Fenton TR (2022) HPV-associated oropharyngeal cancer: epidemiology, molecular biology and clinical management. Nat Rev Clin Oncol May 19(5):306–327. 10.1038/s41571-022-00603-7 10.1038/s41571-022-00603-7 PMC8805140 35105976

[5] Chiesa-Estomba CM, Urazan JD, Cammaroto G et al (2023) Lymph node metastasis in level IIb in oropharyngeal squamous cell carcinoma: a multicentric, longitudinal, retrospective analysis. Eur Arch Otorhinolaryngol Feb 280(2):869–876. 10.1007/s00405-022-07647-6 10.1007/s00405-022-07647-6 36102986

[6] Klug TE, Rusan M, Fuursted K, Ovesen T (2016) Peritonsillar Abscess: Complication of Acute Tonsillitis or Weber’s Glands Infection? Otolaryngol Head Neck Surg Aug 155(2):199–207. 10.1177/0194599816639551 10.1177/0194599816639551 27026737

[7] Long B, Gottlieb M (2023) Managing peritonsillar abscess. Ann Emerg Med 82(1):101–107. 10.1016/j.annemergmed.2022.10.023 36669912 10.1016/j.annemergmed.2022.10.023

[8] Koskinen AI, Hemminki O, Försti A, Hemminki K (2022) Incidence and survival in oral and pharyngeal cancers in Finland and Sweden through half century. BMC Cancer Mar 02(1):227. 10.1186/s12885-022-09337-2 10.1186/s12885-022-09337-2 PMC8889707 35236321

[9] Klug TE (2014) Incidence and microbiology of peritonsillar abscess: the influence of season, age, and gender. Eur J Clin Microbiol Infect Dis Jul 33(7):1163–1167. 10.1007/s10096-014-2052-8 10.1007/s10096-014-2052-8 24474247

[10] Windfuhr J (2002) Malignant neoplasia at different ages presenting as peritonsillar abscess. Otolaryngol Head Neck Surg Feb 126(2):197–198. 10.1067/mhn.2002.122260 10.1067/mhn.2002.122260 11870355

[11] Holmes SB, Vora K, Hardee PS (2001) Squamous cell carcinoma presenting as a peritonsillar abscess. Br J Oral Maxillofac Surg Feb 39(1):46–48. 10.1054/bjom.2000.0567 10.1054/bjom.2000.0567 11178855

[12] Kallel S, Hadj Taieb H, Makni S, Ghorbel A (2013) Lymphoma presenting as a peritonsillar abscess. Eur Ann Otorhinolaryngol Head Neck Dis Dec 130(6):337–339. 10.1016/j.anorl.2012.09.012 10.1016/j.anorl.2012.09.012 23562230

[13] Rokkjaer MS, Klug TE (2015) Tonsillar malignancy in adult patients with peritonsillar abscess: retrospective study of 275 patients and review of the literature. Eur Arch Otorhinolaryngol Sep 272(9):2439–2444. 10.1007/s00405-014-3186-0 10.1007/s00405-014-3186-0 25001851

[14] Xie SH, Santoni G, Gottlieb-Vedi E, Mattsson F, Lagergren J (2025) Swedish Prescribed Drugs and Health Cohort (SPREDH): cohort profile update. BMJ Open Oct 29(10):e104455. 10.1136/bmjopen-2025-104455 10.1136/bmjopen-2025-104455 PMC12574413 41161854

[15] Socialstyrelsen (2021) Det statistiska registrets framställning och kvalitet. Läkemedelsregistret [Internet] [in Swedish]. Stockholm: Socialstyrelsen; [cited 2025 Dec 16]. Available from: https://www.socialstyrelsen.se/globalassets/sharepoint-dokument/dokument-webb/statistik/statistiska-registrets-framstallning-kvalitet-lakemedelsregistret.pdf

[16] Ludvigsson JF, Andersson E, Ekbom A et al (2011) External review and validation of the Swedish national inpatient register. BMC Public Health Jun 09:11:450. 10.1186/1471-2458-11-450 10.1186/1471-2458-11-450 PMC3142234 21658213

[17] Barlow L, Westergren K, Holmberg L, Talbäck M (2009) The completeness of the Swedish Cancer Register: a sample survey for year 1998. Acta Oncol 48(1):27–33. 10.1080/02841860802247664 18767000 10.1080/02841860802247664

[18] eHälsomyndigheten Purchase of medicinal products in Sweden [Internet]. Stockholm: eHälsomyndigheten. [cited 2025 Sep 17]. Available from: https://www.ehalsomyndigheten.se/sprak/english/eu/purchase-of-medicinal-products/

[19] Armitage JN, van der Meulen JH, Group RCoSC-mC (2010) Identifying co-morbidity in surgical patients using administrative data with the Royal College of Surgeons Charlson Score. Br J Surg 97(5):772–781. 10.1002/bjs.6930 20306528 10.1002/bjs.6930

[20] Bergman D, Hagström H, Capusan AJ et al (2020) Incidence of ICD-Based Diagnoses of Alcohol-Related Disorders and Diseases from Swedish Nationwide Registers and Suggestions for Coding. Clin Epidemiol 12:1433–1442. 10.2147/CLEP.S285936 33408530 10.2147/CLEP.S285936 PMC7781026

[21] Läkemedelsverket Läkemedelsboken (2024) Halsinfektioner [Internet] [In Swedish]. Uppsala: Läkemedelsverket; [updated 2024 sep 26: cited 2026 jan 26]. Available from: https://lakemedelsboken.se/terapiomraden/oron--nas--och-halssjukdomar/oron--nas--och-halssjukdomar/halssjukdomar/halsinfektioner/#heading-9509

[22] Risberg S, Engfeldt P, Hugosson S (2008) Incidence of peritonsillar abscess and relationship to age and gender: retrospective study. Scand J Infect Dis 40(10):792–796. 10.1080/00365540802195226 18609198 10.1080/00365540802195226

[23] Sunnergren O, Swanberg J, Mölstad S (2008) Incidence, microbiology and clinical history of peritonsillar abscesses. Scand J Infect Dis 40(9):752–755. 10.1080/00365540802040562 19086341 10.1080/00365540802040562

[24] Gazzini L, Fazio E, Dallari V et al (2022) Impact of the COVID-19 pandemic on head and neck cancer diagnosis: data from a single referral center, South Tyrol, northern Italy. Eur Arch Otorhinolaryngol Jun 279(6):3159–3166. 10.1007/s00405-021-07164-y 10.1007/s00405-021-07164-y PMC8568686 34739577

[25] Lau AS, Milinis K, Roode M et al (2021) The prevalence of oropharyngeal squamous cell carcinoma in patients admitted with symptoms of peritonsillar abscess or cellulitis: A retrospective multicentre study. Clin Otolaryngol Nov 46(6):1362–1367. 10.1111/coa.13851 10.1111/coa.13851 34407287

[26] Creaney G, McMahon AD, Ross AJ, Bhatti LA, Paterson C, Conway DI (2022) Head and neck cancer in the UK: what was the stage before COVID-19? UK cancer registries analysis (2011–2018). Br Dent J Nov 233(9):787–793. 10.1038/s41415-022-5151-4 10.1038/s41415-022-5151-4 PMC9650177 36369569

